# Src family kinases activity is required for transmitting purinergic P2X7 receptor signaling in cortical spreading depression and neuroinflammation

**DOI:** 10.1186/s10194-021-01359-8

**Published:** 2021-12-04

**Authors:** Lingdi Nie, Dongqing Ma, John P. Quinn, Minyan Wang

**Affiliations:** 1 Department of Biological Sciences, Centre for Neuroscience, Xi’an Jiaotong-Liverpool University (XJTLU), 111 Ren Ai Road, Suzhou Industrial Park, Suzhou, 215123 P. R. China; 2 Department of Pharmacology and Therapeutics, Institute of Systems, Molecular and Integrative Biology, Liverpool, L69 7ZB UK

**Keywords:** Migraine, cortical spreading depression, P2X7 receptor, Src family kinases, glutamatergic pathway, neuroinflammation

## Abstract

**Background:**

Purinergic P2X7 receptor plays an important role in migraine pathophysiology. Yet precise molecular mechanism underlying P2X7R signaling in migraine remains unclear. This study explores the hypothesis that P2X7 receptor transmits signaling to Src family kinases (SFKs) during cortical spreading depression (CSD) and neuroinflammation after CSD.

**Methods:**

CSD was recorded using electrophysiology in rats and intrinsic optical imaging in mouse brain slices. Cortical IL-1β and TNFα mRNA levels were detected using qPCR. Glutamate release from mouse brain slices was detected using glutamate assay.

**Results:**

The data showed that deactivation of SFKs by systemic injection of PP2 reduced cortical susceptibility to CSD in rats and CSD-induced IL-1β and TNF-α gene expression in rat ipsilateral cortices. Consistently, in mouse brain slices, inhibition of SFKs activity by saracatinib and P2X7 receptor by A740003 similarly reduced cortical susceptibility to CSD. When the interaction of P2X7 receptor and SFKs was disrupted by TAT-P2X7, a marked reduction of cortical susceptibility to CSD, IL-1β gene expression and glutamate release after CSD induction were observed in mouse brain slices. The reduced cortical susceptibility to CSD by TAT-P2X7 was restored by NMDA, and disrupting the Fyn-NMDA interaction using TAT-Fyn (39-57) but not disrupting Src-NMDA receptor interaction using TAT-Src (40-49) reduced cortical susceptibility to CSD. Furthermore, activation of P2X7 receptor by BzATP restored the TAT-Fyn (39-57)-reduced cortical susceptibility to CSD.

**Conclusion:**

This study reveals that SFKs activity transmits P2X7 receptor signaling to facilitate CSD propagation via glutamatergic pathway and promote neuroinflammation, which is of particular relevance to migraine.

## Highlights

• SFKs activity-mediated P2X7 receptor signaling facilitates CSD propagation via glutamatergic pathway and also promotes neuroinflammation.

• P2X7 receptor/SFKs signaling can be targeted for migraine prophylaxis and therapy.

## Background

Migraine is a neurovascular disorder that is characterized by complicated pathophysiology, among which cortical spreading depression (CSD) is a key event leading to both central and peripheral sensitization [1]. CSD is a temporary propagating wave of depolarization followed by depression in cerebral cortex and subcortical regions [1]. This event disrupts ionic homeostasis and induces release of adenosine triphosphate (ATP) [2] and neurotransmitters especially glutamate [3] and calcitonin gene-related peptide (CGRP) [4], all of which subsequently result in aberrant cortical excitability. CSD also causes neuroinflammation via mast cell degranulation, expression and release of inflammatory cytokines [5, 6] and increases cerebral and meningeal blood flow [7, 8]. All of these result in the activation and sensitization of meningeal nociceptors and trigeminovascular system, which ultimately evoke the onset of migraine headache [9]. More recently, CSD is shown to induce facial hyperalgesia, photophobia and hypomotility in mice, supporting the role of CSD as a nociceptive stimulus underlying migraine with aura [10].

The molecular mechanisms underlying CSD-induced migraine is not fully understood, but activation of membrane receptors and channels, some of which are N-methyl-D-aspartic acid (NMDA) receptors [11], gamma-Aminobutyric acid receptors [12], purinergic P2X7 receptor [13], transient receptor potential ankyrin 1 [14] and Pannexin-1 (Panx1) [7], have been well known to contribute to migraine pathogenesis. Among these receptors, P2X7 receptor, a member of the purinergic receptor family that can be activated in response to ATP, has been increasingly drawn attention in that this receptor regulates a plethora of cellular signaling events in both central and peripheral nervous system [15]. P2X7 receptor possesses an ion channel function and also opens a large transmembrane pore that is permeable to large hydrophilic molecules via forming a complex with Panx1 [16]. In nitroglycerin (NTG)-induced mouse migraine model, inhibition of P2X7 receptor activity attenuates mechanical and thermal hyperalgesia, inflammatory response and central sensitization via promoting autophagic process in trigeminal nucleus caudalis (TNC) [17, 18]; whilst the recurrent NTG in turn increases P2X7 receptor protein expression in TNC [18]. In CSD-induced migraine model, inhibition of P2X7 receptor pore formation attenuates cortical susceptibility to CSD, CSD-induced neuroinflammation and trigeminovascular activation [13]. Despite that P2X7 receptor plays a crucial role in migraine progression, the underlying mechanism by which P2X7 receptor transmits signaling remains unclear.

As an intracellular signaling molecule, Src family kinases (SFKs) have an identified interaction with P2X7 receptor in various cell models. P2X7 receptor physically interacts with SFKs, whilst activation of P2X7 receptor increases phosphorylated SFKs level at tyrosine 416 site in J774 macrophages [19]. Functionally, deactivation of SFKs reduces both P2X7 receptor-induced pore formation [19] and morphine-induced P2X7 receptor-mediated currents and Ca^2+^ responses [20]. SFKs also regulate P2X7 receptor-mediated IL-1β release and reactive oxygen species production via p38 mitogen-activated protein kinase (MAPK) in glial cells [21, 22] and extracellular-signal-regulated kinase 1/2 in macrophage [22] respectively. Given that SFKs activity is required for CSD propagation [23, 24] and is recently proposed as an emerging target for migraine therapy [25], it is likely that SFKs are a key mediator downstream of P2X7 receptor to facilitate migraine progression.

Here we examine the hypothesis that activation of SFKs is required for P2X7 receptor signaling in mediating CSD and CSD-associated cortical neuroinflammation. The involvement of glutamatergic pathway in P2X7 receptor/SFKs signaling during CSD was also explored. Our data identifies that the P2X7 receptor signals to SFKs, the activation of which facilitates CSD propagation and neuroinflammation.

## Methods

### Animals

A total of 35 adult male Sprague-Dawley rats (311.6 ± 6.8 g), 54 adult male C57BL/6J mice (20.9 ± 0.27 g) and 22 adult male Balb/c mice (21.6 ± 0.3 g) were purchased from Shanghai SLAC Laboratory Animal Corporation Ltd. Only the males were used in this study so as to minimize any potential impact of fluctuating levels of hormones in females. Animal procedures were carried out under ethic approval by Xi’an Jiaotong-Liverpool University (XJTLU) in agreement with Soochow University. Animal experiments were performed in accordance with relevant national and provincial guidelines. Randomization was applied for animal use and experimental group setup.

### Induction and recording of CSD by electrophysiology in rats and experimental design

Rats were anesthetized with isoflurane in O_2_: N_2_O (1:2) while breathing spontaneously [24, 26]. The depth of anesthesia was monitored by observing reflexive responses of rats and electroencephalogram (EEG) signals recorded by a digital oscilloscope (DS1000B, RIGOL, Beijing, China) to avoid animal suffering. Two burr holes were drilled on the right skull with dura intact: a posterior hole (coordinate: 5 mm posterior and 2 mm lateral to bregma) was used for CSD induction and an anterior hole (coordinate: 3 mm anterior and 2 mm lateral to bregma) for implanting a Ag/AgCl electrode (0.1 mm, Applied Neuroscience, London, UK), followed by CSD recording. A grounded reference electrode was placed under the scalp and the extracellular direct current (DC) potential was generated between the reference and recording electrodes for recording CSD signal. After stabilization for 1 hour post-surgery, CSD was induced by topical application of 2 M KCl onto the dura [27] for 5 minutes which typically elicits one CSD wave, followed by washing off with artificial cerebrospinal fluid (ACSF, composition in mM: 2.5 NaCl, 250 KCl, 1.18 MgCl_2_, 1.26 CaCl_2_; pH 7.35 - 7.45).

The generated EEG and DC signals were first amplified with a AC/DC pre-amplifier (NL834) and then with a AC/DC amplifier (NL106) to gain 5000 × and 250 × amplification respectively. The EEG signal at 1 - 30 Hz and DC signal at 0 - 30 Hz were collected by a filter (NL125). All electrophysiological equipment were purchased from Digitimer (Welwyn Garden City, UK). All signals were digitized and displayed via Labview 11.0 (National Instruments, Austin, TX, USA). As reported previously [23], CSD latency (duration from KCl application to the start of the rising phase of CSD) and propagation rate were calculated to reflect cortical susceptibility to CSD *in vivo*.

An earlier study reported that inhibition of P2X7 receptor pore complex reduced CSD susceptibility and CSD-induced neuroinflammation in rodents [13]. In this study, we addressed whether systemic deactivation of SFKs could similarly reduce cortical susceptibility to CSD and neuroinflammation after CSD in rats. Previously, we found that deactivation of SFKs by intracerebroventricular (*i.c.v.*) injection of a SFKs inhibitor PP2 reduced cortical susceptibility to CSD in rats [24], suggesting that cortical SFKs activity plays a crucial role in mediating CSD. However, *i.c.v.* injection is not a clinically applicable route of administration and thus could not determine if SFKs inhibitor is a practical treatment for migraineurs. In order to resolve that issue and if peripheral modulation of SFKs activity might also have a role in CSD, we examined if systemic deactivation of SFKs affects CSD in rats by investigating the effects of the SFKs inhibitor, PP2 (1407, Tocris, Bristol, UK) or its negative analog, PP3 (2794, Tocris, Bristol, UK) on CSD and CSD-induced IL-1β and TNFα mRNA levels in rat cortices ipsilateral to CSD. Each rat was administered with respective drug twice via intraperitoneal injection (*i.p.*) for two consecutive days. Surgery was carried out immediately following the 2^nd^ *i.p.* injection for a single CSD induction. Rats were divided into four groups: (i) 2% DMSO (vehicle) without 2 M KCl application; (ii) 2% DMSO with KCl application; (iii) 1mg/kg PP2 with KCl application; (iv) 1mg/kg PP3 with KCl application (*n* = 7 for each). After CSD recording, rats were sacrificed immediately, and ipsilateral cerebral cortices were collected for subsequently detecting IL-1β and TNFα mRNA levels.

### Induction and recording of CSD by intrinsic optical imaging in mouse brain slices and experimental design

The complete procedure of this experiment referred to our previous publication [23]. Briefly, mouse brain slices at 400 μm of thickness were prepared using a vibratome (7000 smz-2, Campden Instruments, Oxford, UK). Each brain slice was placed in a chamber perfused with Kreb’s solution (composition in mM: 126 NaCl, 2.5 KCl, 2.4 CaCl_2_•2H_2_O, 1.3 MgCl_2_•6H_2_O, 18 NaHCO_3_; 1.2 NaH_2_PO_4_, 10 glucose, pH 7.35 – 7.45). CSD was induced on somatosensory cortex by ejection of 260 mM KCl. Intrinsic optical images of the brain slice were continuously recorded for 15 minutes by a monochrome camera (Rolera-XR, ROL-XR-F-M-12, Qimaging, Media Cybernetics, Marlow, UK), which was synchronized with a LED spotlight (SLS-0307-A, Mightex, Pleasanton, CA, USA) via a LED controller (SLC-SA04US, Mightex, Pleasanton, CA, USA). Image Pro Plus software (IPP7.0, Media Cybernetics, Shanghai, China) was used to merge all captured images in which a propagated CSD wave front can be seen. An area of interest (AOI) was selected in the images and a biphasic CSD curve was generated by plotting averaged grey level within the AOI against time. Cortical susceptibility to CSD was represented by CSD latency (the time that takes for a CSD wave propagating to the AOI from the site of KCl ejection) and propagation rate.

Three series of studies were designed using this *ex vivo* CSD model.

Series 1: In order to study whether modulation of SFKs or P2X7 receptor activity could similarly regulate cortical susceptibility to CSD, the effects of a SFKs inhibitor, saracatinib (S1006, Selleckchem, Houston, USA), that is currently investigated in clinical trials for treating several neurological disorders [25, 28] or a P2X7 receptor antagonist, A740003 (3701, Tocris, Bristol, UK) on CSD latency and propagation rate were examined respectively in C57BL/6J mice brain slices. In this series, three groups were designed as follows: (i) 0.03% DMSO (vehicle) (*n* = 8); (ii) 0.5 μM saracatinib (*n* = 8). (iii) 3 μM A740003 (*n* = 8). It should be noted that two P2X7 receptor antagonists, brilliant blue G, a noncompetitive P2X7 receptor antagonist and A438079, a competitive antagonist with higher potency and selectivity [29] were previously shown to reduce cortical susceptibility to CSD in rodents [13]. A740003 was applied in this study as it is also a potent and highly specific P2X7 receptor competitive antagonist [30, 31] that inhibits both the channel activity and pore formation. Additionally, this drug is more effective than A438079 in blocking the P2X7 receptor agonist BzATP-induced IL-1β release and pore formation in human cells [30] and in blocking spinal nerve ligation-induced mechanical allodynia [32].

Series 2: Using the mouse brain slice CSD model, we next investigated whether disruption of P2X7 receptor-SFKs interaction could reduce cortical susceptibility to CSD, neuroinflammation and glutamate release after CSD. TAT-P2X7 (SLHDSPPTPGQGGGYKKRRQRRR) is a peptide that mimics the Src homology 3 (SH3) domain binding site of the COOH terminus of P2X7 receptor, which disrupts the binding between P2X7 receptor and SFKs [19]. This drug specifically blocks P2X7 receptor pore formation without affecting the cation channel activity [33]. In this series, three groups were designed and the peptides concentration were applied by referencing to previously described [34]: (i) Kreb’s (vehicle) (*n* = 8); (ii) 3 μM TAT-P2X7 (*n* = 8); (iii) 3 μM TAT-P2X7SC (HSPLDSPPQTGGGGYKKRRQRRR), the scrambled control of TAT-P2X7 (*n* = 8). C57BL/6J mice carry a spontaneous P451L mutation in the SFKs binding site of their P2X7 receptor gene, causing less SFKs binding and impaired pore formation in these mice. Therefore, in this series, Balb/c instead of C57BL/6J mice were used because Balb/c mice carry a fully functional P2X7 receptor without such mutation [35, 36]. Immediately following CSD recording, the brain slices were collected for measuring IL-1β and TNFα mRNA levels by qPCR and the media in the chamber was collected for measuring glutamate release using glutamate assay.

Series 3: P2X7 receptor exhibits functional interaction with NMDA receptor in neurons and a rat epilepsy model [37–39] while SFKs regulate NMDA receptor activity and function [40]. Since the NMDA receptor is required for CSD propagation [11, 26, 41], we then explored the involvement of the NMDA receptor in SFKs-mediated P2X7 receptor signaling during CSD.

First, we examined if the NMDA receptor agonist, NMDA, could reverse the inhibitory effects of TAT-P2X7 on CSD and neuroinflammation after CSD in Balb/c mice brain slices. Two groups were designed: (i) 3 μM TAT-P2X7 (*n* = 8); (ii) 3 μM TAT-P2X7 + 10 μM NMDA (M3262, Sigma-Aldrich, St. Louis, MO, USA) (*n* = 8). Immediately following CSD recording, the brain slices were collected for measuring IL-1β mRNA level by qPCR.

Next, we tested whether the interaction between NMDA receptor and either of the two SFKs subtypes, Fyn or Src mediates cortical susceptibility to CSD in C57BL/6J mice brain slices. Two peptides that specifically block the binding of Fyn or Src from NMDA receptor complex and the Fyn- or Src-dependent NMDA receptor activity [42–45] were applied: TAT-Fyn (39-57) (YPSFGVTSIPNYNNFHAAGYGRKKRRQRRR) or TAT-Src (40-49) (KPASADGHRGYGRKKRRQRRR) and their scrambled controls, TAT-Fyn (39-57) SC (PSAYGNPGSAYFNFTNVHIYGRKKRRQRRR) or TAT-Src (40-49) SC (GAAKRPSDGHYGRKKRRQRRR). TAT-Fyn (39-57) and TAT-Src (40-49) correspond to the amino acids 39-57 and 40-49 of the unique domain of Fyn and Src respectively [42, 43]. Experiments were designed as follows in this part: (i) Kreb’s (vehicle) (*n* = 7); (ii)1 μM TAT-Fyn (39-57) or 1 μM TAT-Src (40-49) (*n* = 7 for each); (iii) 1 μM TAT-Fyn (39-57) SC or 1 μM TAT-Src (40-49) SC (*n* = 7 for each).

Finally, we studied whether the P2X7 receptor agonist, BzATP could reverse the inhibitory effect of TAT-Fyn (39-57) on CSD in C57BL6/J mice brain slices. Two groups were designed: (i) 1 μM TAT-Fyn (39-57) (*n* = 7); (ii) 1 μM TAT-Fyn (39-57) + 300 μM BzATP (B6396, Sigma-Aldrich, St. Louis, MO, USA) (*n* = 7).

For the above series of experiments, all drugs were applied on brain slices for 1 hour until the end of CSD recording. All peptides were customized from A^+^ peptide (Shanghai, China).

### Quantitative polymerase chain reaction (qPCR)

Total RNA from rat cerebral cortices or mouse brain slices was extracted using TRIZOL reagent (T9424, Sigma-Aldrich, St. Louis, MO, USA), followed by reverse transcription to cDNA using GoScript Reverse Transcription System (A5001, Promega, Madison, WI, USA) in Veriti 96-well Thermal Cycler (Applied Biosystems, Waltham, MA, USA). The levels of IL-1β and TNFα mRNA in rat cerebral cortices or mouse brain slices was detected using GoTaq qPCR Master Mix (A6002, Promega, Madison, WI, USA) in QuantStudio 5 Real-Time PCR System (Applied Biosystems, Waltham, MA, USA). Two housekeeping genes, peptidylprolyl isomerase A (PPIA) and β-actin (ACTB), were used for comparative analysis. Primers used to target these genes were as follows: rat IL-1β forward 5’GCTGTGGCAGCTACCTATGTCTTG3’, reverse 5’AGGTCGTCATCATCCCACGAG3’; mouse IL-1β forward 5’ACTACAGGCTCCGAGATGAACAAC3’, reverse 5’CCCAAGGCCACAGGTATTTT3’; rat TNFα forward 5’CGGGCTCAGAATTTCCAACA3’, reverse 5’CGCAATCCAGGCCACTACTT3’; mouse TNFα forward 5’TGCACCACCATCAAGGACTCAAAT3’, reverse 5’ CCCCGGCCTTCCAAATAAATACAT3’; rat/mouse ACTB forward 5’CTGTCCACCTTCCAGCAGAT3’, reverse 5’CGCAGCTCAGTAACAGTCCG3’; rat/mouse PPIA forward 5’TTGCTGCAGACATGGTCAAC3’, reverse 5’TGTCTGCAAACAGCTCGAAG3’. The levels of IL-1β or TNFα mRNA were normalized to the geometric mean of the mRNA levels of the two housekeeping genes.

### Glutamate assay

The level of glutamate released into the media submerging mouse brain slices was measured using a Glutamate Assay Kit (ab83389, Abcam, Cambridge, UK). Briefly, the media and a series of diluted standard solutions were added into a clear assay plate, followed by incubating with glutamate developer and glutamate enzyme mix at 37°C for 30 minutes. The optical density (OD) at 450 nm was read using a colorimetric microplate reader (BioTek, Winooski, VT, USA). A standard curve correlating the OD value to the concentration of glutamate in the standard solutions was generated to calculate the concentration of glutamate in the media.

### Statistical analysis

Data were analyzed using GraphPad Prism 7.0. A normality test was performed for all data by Shapiro-Wilk test. For comparison between two independent groups, if the data fulfilled normality, they were presented as mean ± standard error of the mean and analyzed by two-tailed unpaired t-test; if not, they were presented as median (interquartile range) and analyzed by two-tailed Mann-Whitney test. Significant differences were indicated by * *p* < 0.05, ** *p* < 0.01, *** *p* < 0.001, **** *p* < 0.0001

## Results

### Systemic inhibition of SFKs activity reduced cortical susceptibility to CSD and CSD-associated neuroinflammation in rat ipsilateral cortices

**Fig. 1:**
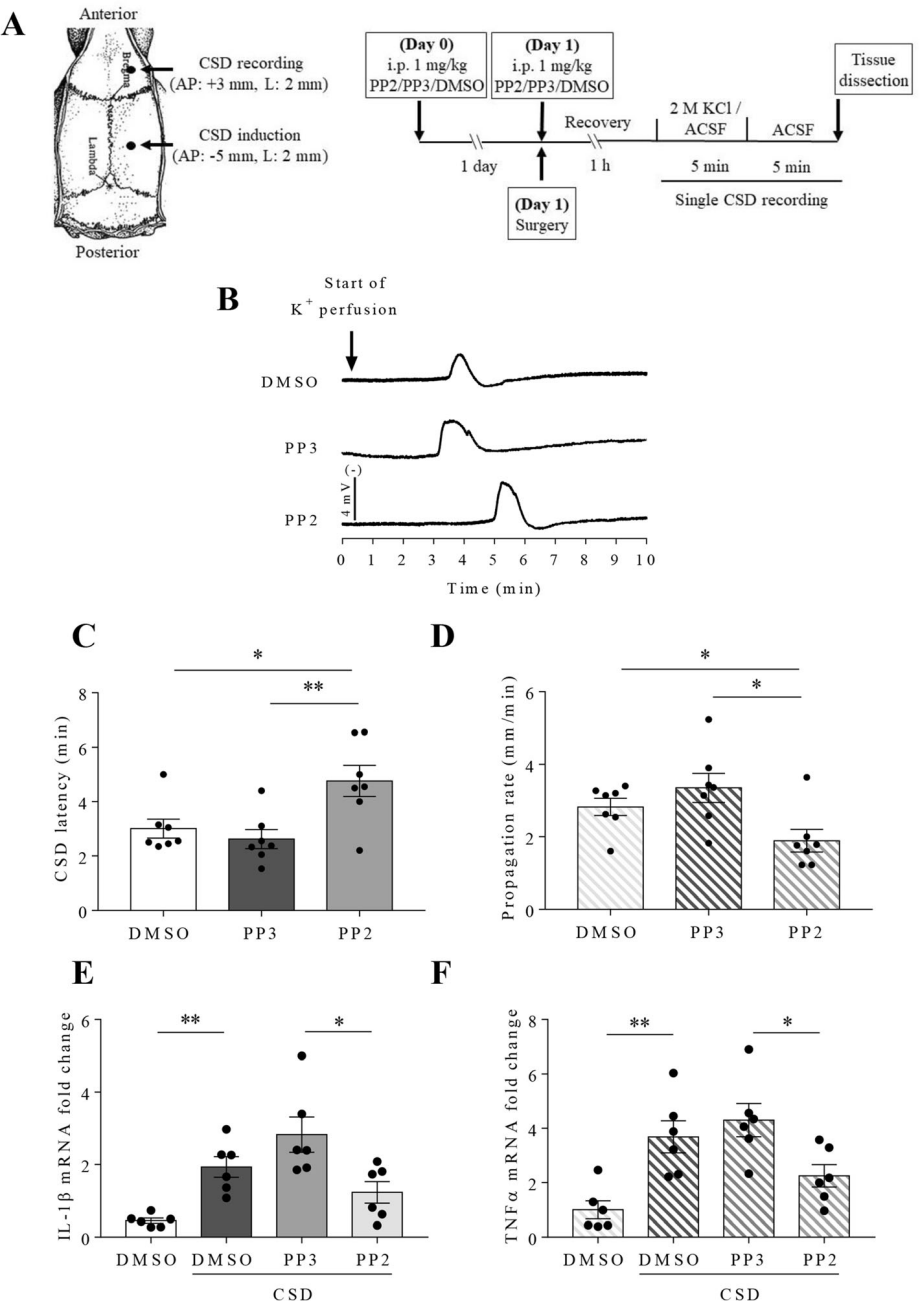
Systemic deactivation of SFKs reduced cortical susceptibility to CSD and CSD-associated neuroinflammation in rat ipsilateral cortices. **a** Schematic presentation of cranial preparation and *in vivo* experimental protocol. **b** Representative traces of CSD affected by 1mg/kg PP2 and PP3. **c d** Effects of 2% DMSO, 1 mg/ml PP2 and PP3 on CSD latency and propagation rate. **e f** Effects of 2% DMSO, 1 mg/ml PP2 and PP3 on IL-1β and TNFα mRNA fold change after CSD in rat ipsilateral cerebral cortices. Two-tailed unpaired t-test was used for comparison in CSD latency and propagation rate between DMSO and PP2 group, PP2 and PP3 group; in IL-1β and TNFα mRNA fold change between DMSO without CSD and DMSO with CSD group, DMSO and PP2 with CSD group, PP2 and PP3 with CSD group. Significant differences were indicated by * *p* < 0.05, ** *p* < 0.01

Similar to the reduced cortical susceptibility to CSD under P2X7 receptor inhibition in both rats and mice as reported previously [13], pre-treatment of 1 mg/ml PP2 via *i.p.* injection significantly prolonged CSD latency to 4.8 ± 0.6 minutes when compared to that at 3 ± 0.4 minutes in DMSO group (*p* = 0.0257) and at 2.6 ± 0.3 minutes in the negative control PP3 group (*p* = 0.0095) respectively (*n* = 7 per group) (Fig. 1c). In contrast, PP2 decreased CSD propagation rate to 1.9 ± 0.3 mm/minute when compared to that at 2.8 ± 0.2 mm/minute in DMSO group (*p* = 0.0359) and at 3.4 ± 0.4 mm/minute in PP3 group (*p* = 0.0149) respectively (*n* = 7 per group) (Fig. 1d). Unlike PP2, PP3 did not alter both CSD latency and propagation rate when compared with that in DMSO group.

We next examined if pretreatment of SFKs inhibitor reduces CSD-associated pro-inflammatory cytokines IL-1β and TNFα mRNA levels in rat ipsilateral cerebral cortices. CSD markedly increased both IL-1β and TNFα mRNA to 1.9 ± 0.3 (fold change, *p* = 0.0029) and 3.7 ± 0.6 (*p* = 0.0042) respectively compared to their levels at 0.5 ± 0.1 and 1 ± 0.3 in the absence of CSD. Pretreatment of PP2 attenuated both IL-1β and TNFα mRNA induced by CSD to 1.2 ± 0.3 and 2.2 ± 0.4 when compared to their levels at 2.8 ± 0.5 (*p* = 0.0232) and 4.3 ± 0.6 (*p* = 0.0217) in PP3 group (*n* = 6 per group, Fig. 1e, f). It is noted that although a declining trend of either IL-1β or TNFα mRNA level was seen in PP2 group compared to DMSO group after CSD, the difference was insignificant. Since these levels in PP3 group did not differ from that in DMSO group either, these data support that PP2 but not PP3 affects CSD-associated IL-1β and TNFα mRNA expression.

### Inhibition of P2X7 receptor and SFKs activity reduced cortical susceptibility to CSD in mouse brain slices

**Fig. 2:**
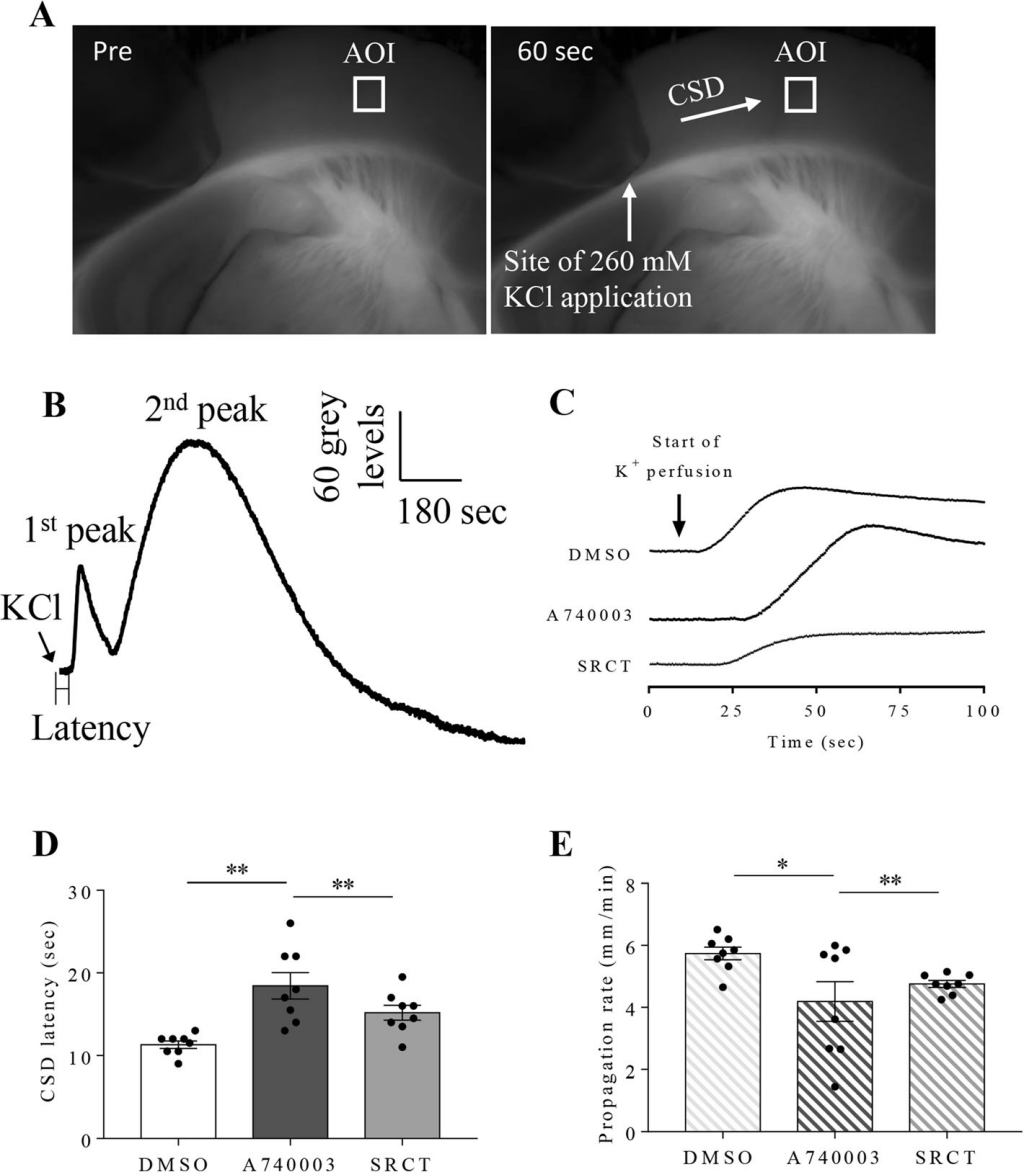
Both deactivation of P2X7 receptor and SFKs reduced cortical susceptibility to CSD in mouse brain slices. **a** The captured images of a mouse brain slice before and after CSD induction by 260 mM KCl in cerebral cortex. The arrow indicated the direction of CSD propagation. An AOI was selected and kept the same for data analysis. **b** The biphasic CSD curve generated from the images recorded for 15 minutes by plotting averaged grey level within the AOI against time. CSD latency (sec) is the time interval between KCl application and CSD elicitation at the AOI. CSD propagation rate (mm/min) is the velocity by which CSD propagates along cerebral cortex. **c** Representative traces of the 1st peak of CSD affected by 0.03% DMSO, 3 μM A740003 and 0.5 μM saracatinib. Only the trace recorded during the first 100 seconds was displayed here in order to clearly show the starting points of KCl application and CSD elicitation. **d e** Effects of 0.03% DMSO, 3 μM A740003 and 0.5 μM saracatinib on CSD latency and propagation rate. Abbreviations: saracatinib (SRCT); seconds (sec); mm/minute (mm/min). Two-tailed unpaired t-test was used for comparison in CSD latency and propagation rate between DMSO and A740003 group, DMSO and saracatinib group. Significant differences were indicated by * *p* < 0.05, ** *p* < 0.01

We investigated the effects of a highly specific P2X7 receptor competitive antagonist A740003 [31, Honore, 2006 #7] or a clinically relevant SFKs inhibitor saracatinib on CSD latency and propagation rate in mouse brain slices. The results demonstrated that perfusion of 3 μM A740003 significantly prolonged CSD latency to 18.4 ± 1.6 seconds and reduced CSD propagation rate to 4.2 ± 0.6 mm/minute when compared to those at 11.31 ± 0.4 seconds (*p* = 0.0026) and 5.7 ± 0.2 mm/minute (*p* = 0.0479) respectively in DMSO group (*n* = 8 per group, Fig. 2d, e). Similar as A740003, albeit to a slightly lesser extent, 0.5 μM saracatinib also prolonged CSD latency to 15.2 ± 0.9 seconds (*p* = 0.0031) and reduced CSD propagation rate to 4.8 ± 0.1 mm/minute (*p* = 0.0014) compared to those in DMSO group (*n* = 8 per group, Fig. 2d, e).

### Disrupting P2X7 receptor-SFKs interaction reduced cortical susceptibility to CSD and CSD-associated neuroinflammation in mouse brain slices

**Fig. 3:**
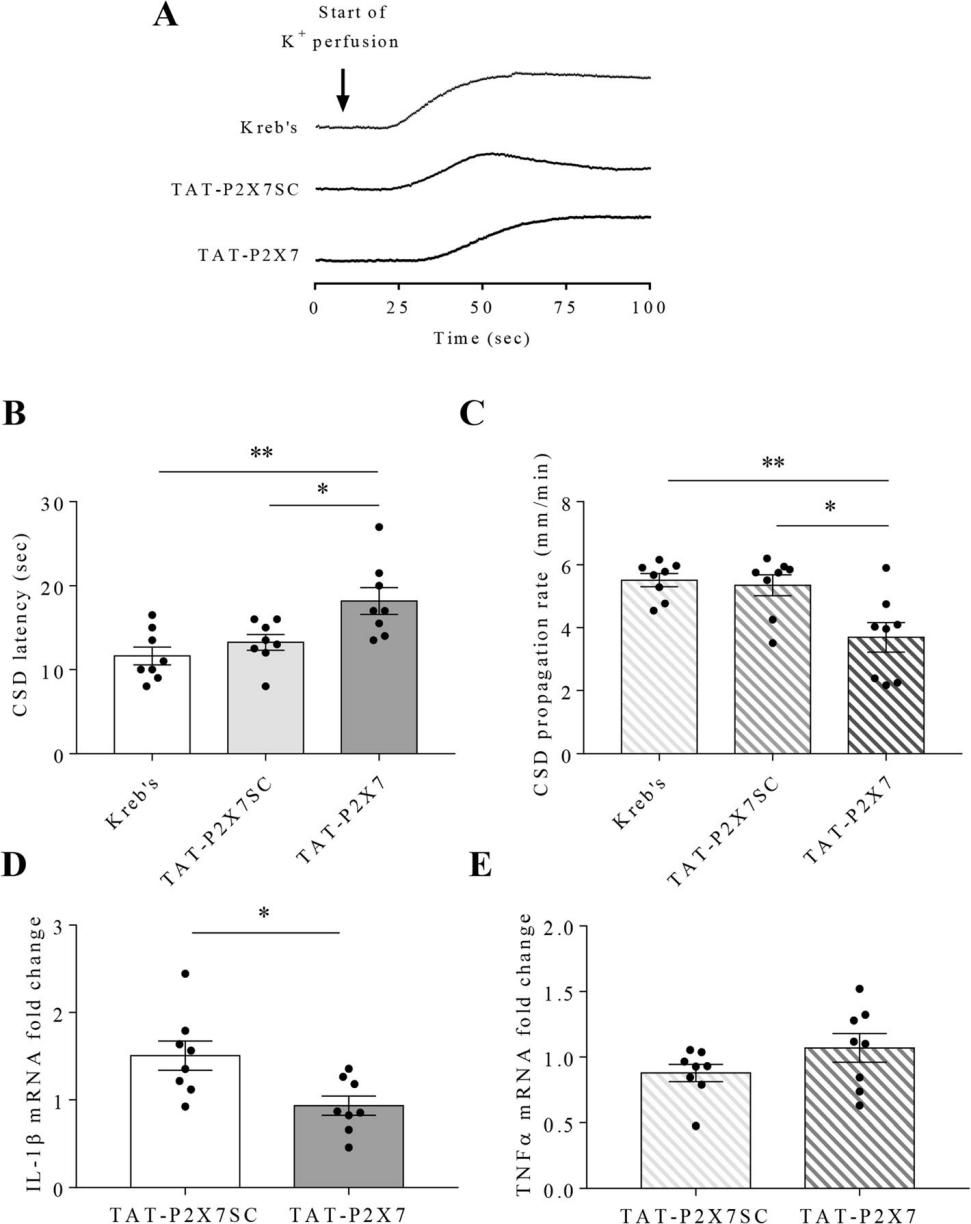
Disrupting P2X7 receptor-SFKs interaction reduced cortical susceptibility to CSD and CSD-associated neuroinflammation in mouse brain slices. **a** Representative traces of 1^st^ peak of CSD affected by Kreb’s, 3 μM TAT-P2X7 and TAT-P2X7SC. Only the trace recorded during the first 100 seconds was displayed here in order to clearly show CSD latency in respective group. **b c** Effects of Kreb’s, 3 μM TAT-P2X7 and TAT-P2X7SC on CSD latency and propagation rate. **d e** Effects of 3 μM TAT-P2X7 and TAT-P2X7SC on IL-1β and TNFα mRNA fold change after CSD in mouse brain slices. Two-tailed unpaired t-test was used for comparison in CSD latency and propagation rate between Kreb’s and TAT-P2X7 group, TAT-P2X7 and TAT-P2X7SC group; in IL-1β and TNFα mRNA fold change between TAT-P2X7 and TAT-P2X7SC group. Significant differences were indicated by * *p* < 0.05, ** *p* < 0.01

We explored whether disrupting the interaction between P2X7 receptor and SFKs affects CSD in mouse brain slices. Perfusion of 3 μM TAT-P2X7 markedly increased CSD latency to 18.2 ± 1.6 seconds and decreased CSD propagation rate to 3.7 ± 0.5 mm/minute in comparison with those at 11.6 ± 1.1 seconds (*p* = 0.0049) and 5.5 ± 0.2 mm/minute (*p* = 0.0058) in Kreb’s group and at 13.3 ± 0.9 seconds (*p* = 0.0209) and 5.3 ± 0.3 mm/minute (*p* = 0.0137) in the scrambled control TAT-P2X7SC group respectively (*n* = 8 per group, Fig. 3b, c).

Consistent to the reduced cortical susceptibility to CSD, 3 μM TAT-P2X7 also attenuated IL-1β mRNA to 0.9 ± 0.1 (fold change, *p* = 0.0144) compared to that at 1.5 ± 0.2 in TAT-P2X7SC group after CSD in mouse brain slices (*n* = 8 per group, Fig. 3d, e). Unlike IL-1β mRNA, CSD-associated TNFα mRNA expression was not altered by TAT-P2X7, the fold change of which were 1.1 ± 0.1 and 0.9 ± 0.1 (*p* = 0.1612) in TAT-P2X7 and TAT-P2X7SC groups respectively.

### Disrupting P2X7 receptor-SFKs interaction reduced CSD-associated glutamate release in mouse brain slices

**Fig. 4:**
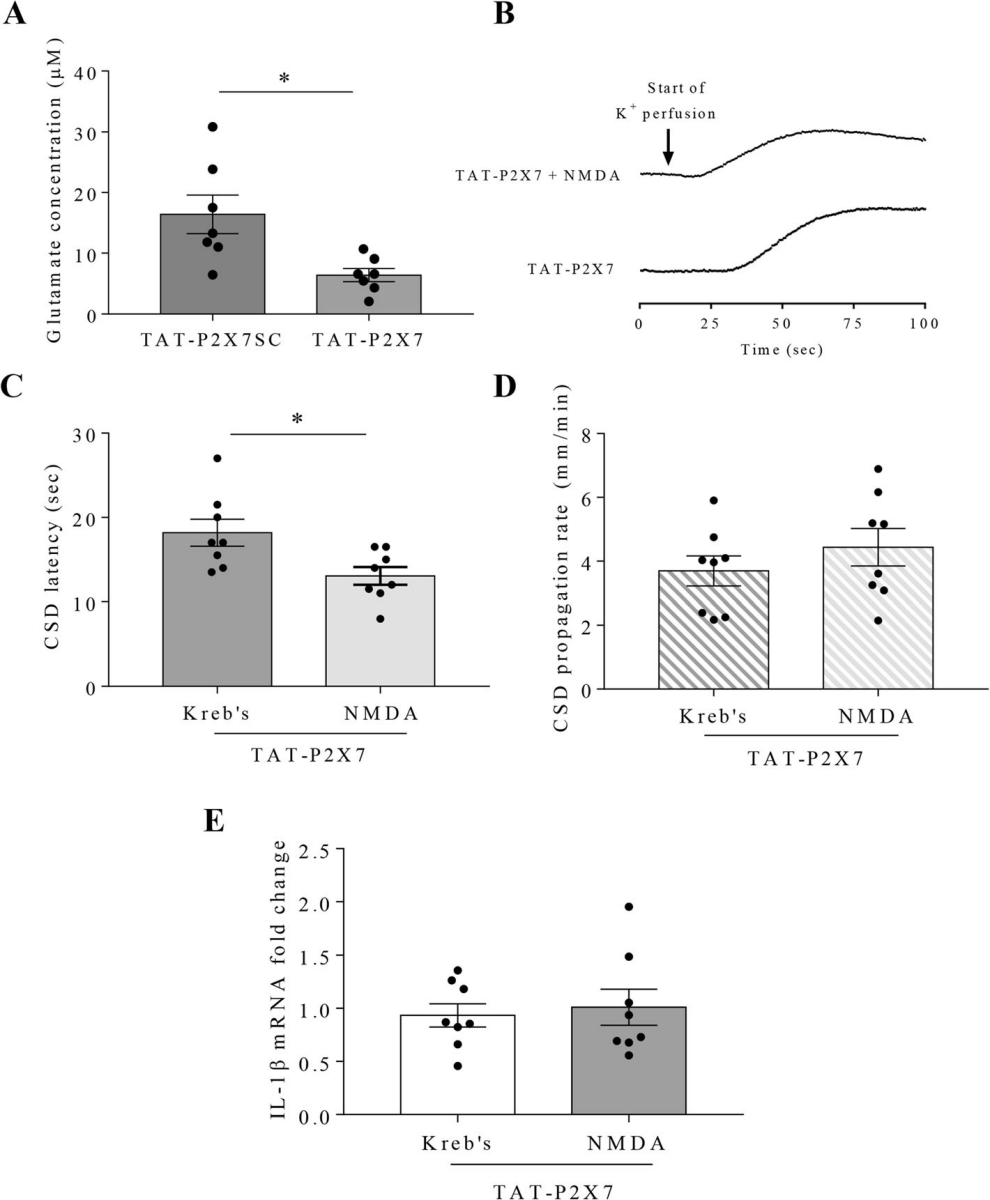
NMDA restored the disrupted P2X7 receptor-SFKs interaction-reduced cortical susceptibility to CSD but not CSD-associated neuroinflammation in mouse brain slices. **a** Effects of 3 μM TAT-P2X7 and TAT-P2X7SC on glutamate release from mouse brain slices after CSD. **b** Representative traces of 1^st^ peak of CSD affected by 3 μM TAT-P2X7 and 3 μM TAT-P2X7 + 10 μM NMDA. **c d** Effects of 3 μM TAT-P2X7 + 10 μM NMDA on CSD latency and propagation rate. **e** Effects of 3 μM TAT-P2X7 + 10 μM NMDA on CSD-associated IL-1β mRNA fold change in mouse brain slices. Two-tailed unpaired t-test was used for comparison in glutamate release between TAT-P2X7 and TAT-P2X7SC group; in CSD latency, propagation rate and IL-1β mRNA fold change between TAT-P2X7 and TAT-P2X7 + NMDA group. Significant differences were indicated by * *p* < 0.05

We next examined whether disruption of P2X7 receptor-SFKs interaction reduces glutamate release after CSD in mouse brain slices. As expected, 3 μM TAT-P2X7 significantly attenuated the level of glutamate released from mouse brain slices after CSD to 6.4 ± 1.1 μM (*p* = 0.0194), which was lower than that at 16.4 ± 3.2 μM in TAT-P2X7SC group (*n* = 7 per group, Fig. 4a).

### NMDA reversed the reduced cortical susceptibility to CSD but not CSD-associated neuroinflammation by TAT-P2X7 in mouse brain slices

We explored the mechanism underlying how P2X7 receptor/SFKs pathway mediates CSD by investigating the involvement of NMDA receptor in this pathway. We first studied whether NMDA application could reverse the reduced cortical susceptibility to CSD and CSD-associated neuroinflammation by TAT-P2X7 in mouse brain slices. Perfusion of 10 μM NMDA restored the CSD latency prolonged by 3 μM TAT-P2X7 from 18.2 ± 1.6 seconds to 13.1 ± 1.1 seconds (*p* = 0.0195, Fig. 4c). Differently, NDMA did not alter the reduced CSD propagation rate by 3 μM TAT-P2X7 (3.7 ± 0.5 mm/minute in TAT-P2X7 group vs. 4.4 ± 0.6 mm/minute in TAT-P2X7 + NMDA group, *p* = 0.3411, *n* = 8 per group, Fig. 4d). Furthermore, NMDA did not affect the reduced IL-1β mRNA fold change by 3 μM TAT-P2X7 (0.9 ± 0.1 in TAT-P2X7 group vs. 1 ± 0.2 in TAT-P2X7 + NMDA group, *p* = 0.7088, *n* = 8 per group, Fig. 4e).

### Disrupting NMDA receptor-Fyn but not -Src interaction reduced cortical susceptibility to CSD in mouse brain slices

**Fig. 5:**
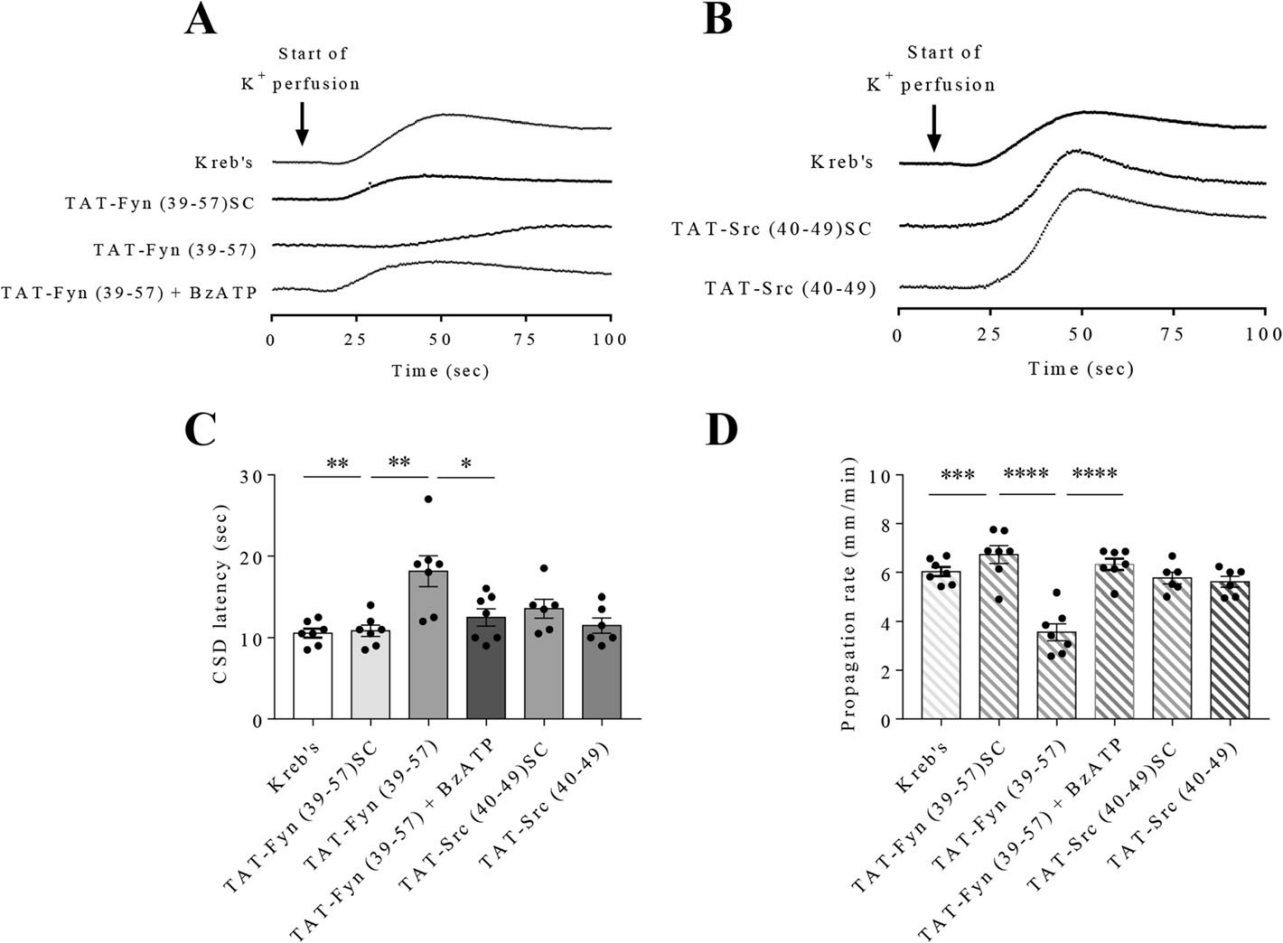
Disrupting NMDA receptor-Fyn but not -Src interaction reduced cortical susceptibility to CSD in mouse brain slices. **a b** Representative traces of 1^st^ peak of CSD affected by Kreb’s, 1 μM TAT-Fyn (39-57), 1 μM TAT-Fyn (39-57) SC, 1 μM TAT-Fyn (39-57) + 300 μM BzATP, 1 μM TAT-Src (40-49) and 1 μM TAT-Src (40-49) SC. **c d** Effects of Kreb’s, 1 μM TAT-Fyn (39-57), 1 μM TAT-Fyn (39-57) SC, 1 μM TAT-Fyn (39-57) + 300 μM BzATP, 1 μM TAT-Src (40-49) and 1 μM TAT-Src (40-49) SC on CSD latency and propagation rate. Two-tailed unpaired t-test was used for comparison in CSD latency and propagation rate between Kreb’s and TAT-Fyn (39-57) group, TAT-Fyn (39-57) and TAT-Fyn (39-57) SC group, TAT-Fyn (39-57) and TAT-Fyn (39-57) + BzATP group, Kreb’s and TAT-Src (40-49) group, TAT-Src (40-49) and TAT-Src (40-49) SC group. Significant differences were indicated by * *p* < 0.05, ** *p* < 0.01, *** *p* < 0.001, **** *p* < 0.0001

We next examined whether disrupting NMDA receptor-Fyn or -Src interaction affects CSD in mouse brain slices. The results showed that 1 μM TAT-Fyn (39-57) significantly prolonged CSD latency to 18.1 ± 1.9 seconds and reduced CSD propagation rate to 3.6 ± 0.3 mm/minute in comparison with that at 10.6 ± 0.6 seconds (*p* = 0.0064) and 6 ± 0.2 mm/minute (*p* = 0.0001) in Kreb’s group and at 10.9 ± 0.7 seconds (*p* = 0.0076) and 6.7 ± 0.4 mm/minute (*p* < 0.0001) in the scrambled control TAT-Fyn (39-57) SC group respectively (*n* = 7 per group, Fig. 5c, d). Unlike TAT-Fyn (39-57), 1 μM TAT-Src (40-49), however, did not affect CSD latency and CSD propagation rate compared to those in both Kreb’s group and TAT-Src (40-49) SC group, the data of which were 11.5 ± 0.9 seconds and 5.6 ± 0.2 mm/minute in TAT-Src (40-49) group and 13.6 ± 1.2 seconds (*p* = 0.1969) and 5.8 ± 0.2 mm/minute (*p* = 0.6507) in TAT-Src (40-49) SC group respectively (n = 6 per group, Fig. 5c, d).

### BzATP reversed the reduced cortical susceptibility to CSD by TAT-Fyn (39-57) in mouse brain slices

We further studied whether activation of P2X7 receptor could restore the reduced cortical susceptibility to CSD by TAT-Fyn (39-57) in mouse brain slices. 300 μM BzATP restored the prolonged CSD latency and the reduced CSD propagation rate by 1 μM TAT-Fyn (39-57) from 18.2 ± 1.6 seconds and 3.7 ± 0.5 mm/minute to 12.5 ± 1.1 seconds (*p* = 0.028) and 6.3 ± 0.2 mm/minute (*p* < 0.0001) respectively in mouse brain slices (n = 7 per group, Fig. 5c, d).

## Discussion

We show for the first time that SFKs activity is required for P2X7 receptor signaling during CSD propagation and CSD-associated neuroinflammation.

Similar as pharmacological inhibition of P2X7 receptor *in vivo* [13], pretreatment of the SFKs inhibitor PP2 via *i.p.* administration suppresses cortical susceptibility to CSD in rats (Fig. 1c, d). This data is in compatible with our previous finding that SFKs regulate cortical susceptibility to CSD in rats, which is shown via *i.c.v.* administration of the same inhibitor PP2 [24]. Consistent to the *in vivo* data, in mouse brain slices, inhibition of both P2X7 receptor by the highly specific P2X7 receptor antagonist A740003 and SFKs activity by the clinically relevant SFKs inhibitor saracatinib showed similar reduction in cortical susceptibility to CSD (Fig. 2d, e). Interestingly, when the interaction between P2X7 receptor and SFKs is disrupted by a peptide TAT-P2X7 that is known to reduce the activated SFKs bound to P2X7 receptor and P2X7 receptor activation-induced SFKs activation [19], then prolonged CSD latency and reduced CSD propagation rate were also observed (Fig. 3b, c). Collectively, these findings suggest an interactive relationship between SFKs and P2X7 receptor in mediating cortical susceptibility to CSD.

Notably, TAT-P2X7 only inhibits P2X7 receptor activation induced-pore formation but not P2X7 receptor channel activity [33]. Hence, the fact that TAT-P2X7 reduces cortical susceptibility to CSD suggests that SFKs participate in P2X7 receptor pore formation to promote CSD propagation. Indeed, inhibition of SFKs activity is previously reported to reduce P2X7 receptor activation-induced cell membrane permeabilization [19]. Consistent with this evidence, selective targeting of P2X7 receptor pore formation by Brilliant blue FCF reduces CSD susceptibility, which is not seen when selective targeting of P2X7 receptor channel by calmidazolium [13]. Additionally, C57BL6/J mice with P451L mutation in their P2X7 receptor and impaired pore formation show lower CSD susceptibility than that of Balb/c mice with fully functional P2X7 receptor [13]. Taken together, our data pinpoint the significant role of SFKs in regulating P2X7 receptor, mainly its pore formation, during CSD propagation.

CSD is known to robustly promote cortical inflammatory cytokines IL-1β an TNFα gene and protein expression [5, 6, 46], leading to neuroinflammation. Inhibition of P2X7 receptor pore formation is previously shown to attenuate CSD-induced cortical IL-1β mRNA expression in rodents [13]. Similarly, in the present study, pretreatment of the SFKs inhibitor also downregulates CSD-induced ipsilateral cortical IL-1β and TNFα mRNA levels in rats (Fig. 1e, f). Interestingly, perfusion of TAT-P2X7 also reduces IL-1β mRNA level after CSD induction in mouse brain slices (Fig. 3d), supporting that disruption of P2X7 receptor-SFKs interaction reduces cortical IL-1β gene expression after CSD. These data demonstrate that P2X7 receptor/SFKs signaling contributes to neuroinflammation associated with CSD. It is noted that IL-1β mRNA transcript and protein are well correlated [47, 48]. Future work could be considered to study if the protein level of IL-1β could be regulated by P2X7 receptor/SFKs signaling at longer time points post-CSD.

The mechanism of P2X7 receptor/SFKs signaling governing CSD-induced IL-1β gene expression is unclear. Interestingly, a recent finding shows that NF-κB p65 co-expresses with P2X7 receptor in mice cortical and subcortical structures. In this study, the authors report that optogenetically-triggered CSD induces NF-κB p65 nuclear translocation, which can be reversed by a P2X7 receptor antagonist [49]. Consistently, Fyn kinase activity also enhances NF-κB p65 nuclear translocation, which correlates with IL-1β gene expression in a Parkinson’s disease model [50]. In the present study, SFKs transmit signaling of P2X7 receptors to mediate CSD (Fig. 3b, c) and CSD-induced IL-1β gene expression (Fig. 3d). Thus, it is likely that the mechanism of P2X7 receptor/SFKs signaling governing CSD-induced IL-1β gene expression may involve NFκB p65 nuclear translocation; however, this model requires future clarification. It is noted that, unlike inhibition of SFKs activity alone in rats (Fig. 1f), TAT-P2X7 does not alter CSD-induced TNFα gene expression in mouse brain slices (Fig. 3e). It is possible that partial SFKs are still active to promote TNFα gene expression via alternative pathways independent of P2X7 receptor.

Albeit the mechanism underlying how SFKs mediate P2X7 receptor signaling during CSD is not fully known, the present study shows involvement of glutamatergic pathway in this process. P2X7 receptor is known to regulate glutamate release from astrocytes and central brain regions, facilitating glia-neuron communication and the development of neuropathic pain [51, 52]. Here, we show that disruption of P2X7 receptor-SFKs interaction by TAT-P2X7 reduces glutamate release from mouse brain slices after CSD (Fig. 4a). Reciprocally, the TAT-P2X7-reduced cortical susceptibility to CSD can be restored by NMDA (Fig. 4c, d). This is consistent with previous findings that both P2X7 receptor and SFKs have functional interaction with NMDA receptor in different models of neurological diseases [37, 38, 40]. Notably, the functional interaction between NR2A-containing NMDA receptor and SFKs mediates CSD as evidenced by a SFK activator, pYEEI, that restored the inhibitory effect of NR2A-containining NMDA receptor antagonist on CSD in mouse brain slices [24]. These data suggest that P2X7 receptor/SFKs signaling-mediated CSD is likely to be dependent on NMDA receptor activity. Unexpectedly, NMDA does not affect the TAT-P2X7-reduced IL-1β mRNA expression after CSD (Fig. 4e), indicating that NMDA receptor may not be involved in the downstream neuroinflammation after CSD in the P2X7 receptor/SFK signaling.

It is noted that disrupting the interaction between NMDA receptor and Fyn by TAT-Fyn (39-57) reduces cortical susceptibility to CSD (Fig. 5c, d). These data imply that Fyn-dependent NMDA receptor activity is required for mediating CSD susceptibility. Unlike disrupting the NMDA receptor-Fyn interaction, disrupting the NMDA receptor-Src interaction by TAT-Src (40-49) does not affect CSD (Fig. 5c, d). As mentioned above, TAT-Fyn (39-57) or TAT-Src (40-49) specifically blocks the binding of Fyn or Src from NMDA receptor complex and the Fyn- or Src-dependent NMDA receptor activity [42, 43]. Therefore, these data support that Fyn but not Src is required for NMDA receptor-mediated CSD propagation. Although we couldn’t exclude the possibility that higher concentration of TAT-Src might have a larger effect, applying the same concentration of the two peptides would generate comparable effects between them. Given that TAT-Fyn (39-57) and TAT-Src (40-49) target the unique domain of Fyn and Src respectively and their structural similarity, the fact that TAT-Fyn exerts more significant effect than TAT-Src at the same concentration might suggest that Fyn plays a more important role than Src in regulating CSD. In order to confirm the involvement of P2X7 receptor in Fyn-NMDA receptor interaction-mediated cortical susceptibility to CSD, we further show that activation of P2X7 receptor by BzATP restores TAT-Fyn (39-57)-reduced cortical susceptibility to CSD (Fig. 5c, d). These findings suggest that SFKs-transmitted P2X7 receptor signaling mediates CSD susceptibility via glutamatergic pathway. Taken together the above evidence and given that NMDA receptor facilitates CSD initiation and propagation [1, 26], we propose that P2X7 receptor/SFKs signaling promotes cortical susceptibility to CSD and CSD-associated glutamate release, which subsequently may sustain NMDA receptor activity and create a positive feedback loop, contributing to CSD-associated migraine pathogenesis.

The mechanism underlying SFKs-mediated P2X7 receptor signaling during CSD is also likely to involve Panx1. Our previous study demonstrates that CSD promotes Panx1-SFKs coupling in cerebral cortices and the interaction between Panx1 and SFKs regulates CSD susceptibility [23]. Similar to P2X7 receptor, SFKs activity also regulates Panx1 activation and channel opening induced by CSD [23]. SFKs are possibly the intermediate protein linking P2X7 receptor and Panx1 to form a functional pore, but whether the three proteins form a complex requires further study. Additionally, SFKs often phosphorylate their interacting proteins to regulate their functions and activities. A more recent study identified tyrosine 382-384 within the second transmembrane domain and the intracellular C-terminus of P2X7 receptor as potential tyrosine phosphorylation sites [20]. Whether SFKs can phosphorylate these sites of P2X7 receptor to regulate P2X7 receptor function and signaling awaits to be studied.

A few limitations exist in this study. It should be noted that this study is carried out only in male rodents as it does in majority of studies investigating the roles of P2X7 receptor [13, 17, 18] and SFKs in migraine models [24, 53] in order to exclude the effect of hormonal fluctuation. However, as migraine affects more women than men, and hormonal fluctuation is a migraine trigger [54], it is necessary to explore the effect of P2X7 receptor/SFKs signaling in CSD in females. In fact, one study explores the role of SFKs in the FHM2 model using equal number of male and female mice and finds no sex difference in the study [55]. The gender specificity of P2X7 receptor is also previously investigated in amyotrophic lateral sclerosis mice model in which different P2X7 receptor antagonists show better efficacy in attenuating disease progression in one sex than the other [56, 57]. Nevertheless, future work is needed to investigate the gender-specific effect of P2X7 receptor/SFKs signaling in migraine models. Another limitation is that dose-response experiments of drugs used in this study are not carried out. Nevertheless, the concentration of respective drug was carefully selected following our preliminary experiments on the basis of respective drug selectivity and the effective concentration range reported in the literature, as cited in the methods section, in an attempt to ensure the selectivity and efficacy of drugs applied in our models.

## Conclusion

**Fig. 6:**
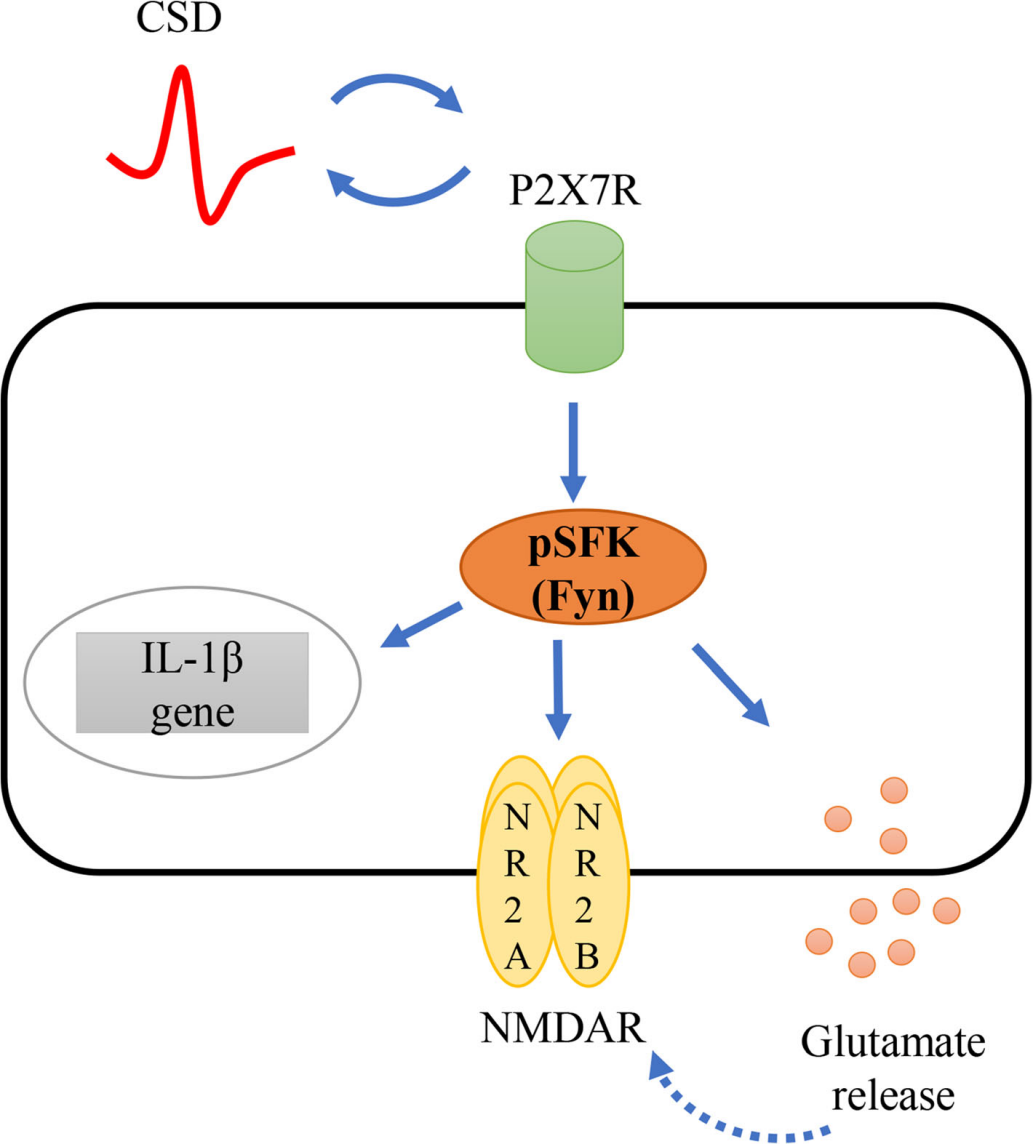
Schematic representation of the role that P2X7 receptor/SFKs signaling may exert in CSD-associated migraine pathophysiology. P2X7 receptor/SFKs signaling is activated during CSD to facilitate neuroinflammation, NMDA receptor activation and glutamate release. Glutamate may in return reinforce the activation of NMDA receptor (dotted line with arrow), both of which facilitate cortical susceptibility to CSD, forming a positive loop

To summarize, our data reveal that SFKs activity mediates P2X7 receptor signaling during CSD propagation and CSD-associated neuroinflammation. We propose a novel SFKs-transmitted P2X7 signaling that facilitates CSD propagation via glutamatergic pathway and CSD-associated neuroinflammation (Fig. 6). Our findings provide evidence for potential clinical application of drugs targeting P2X7 receptor/SFKs signaling in migraine prophylaxis and therapy.

## Data Availability

Data reported in this manuscript are available within the article. Raw data materials are available with the Corresponding author, which can be readily accessed by the Journal upon request.

## Abbreviations

CSD: Cortical spreading depression
SFKs: Src family kinases
NMDA: N-methyl-D-aspartate (NMDA)
CGRP: Calcitonin gene-related peptide
PACAP: Pituitary adenylate-cyclase-activating polypeptide
Panx1: Pannexin 1
NTG: Nitroglycerin
TNC: Trigeminal nucleus caudalis
DC: Direct current
EEG: Electroencephalogram
